# High-resolution cryo-EM using a common LaB_6_ 120-keV electron microscope equipped with a sub–200-keV direct electron detector

**DOI:** 10.1126/sciadv.adr0438

**Published:** 2025-01-03

**Authors:** Hariprasad Venugopal, Jesse Mobbs, Cyntia Taveneau, Daniel R. Fox, Ziva Vuckovic, Sahil Gulati, Gavin Knott, Rhys Grinter, David Thal, Stephen Mick, Cory Czarnik, Georg Ramm

**Affiliations:** ^1^ Ramaciotti Centre for Cryo-Electron Microscopy, Monash University, Clayton, VIC, Australia.; ^2^ Drug Discovery Biology, Monash Institute of Pharmaceutical Sciences, Monash University, Parkville, VIC, Australia.; ^3^ Australian Research Council Centre for Cryo-Electron Microscopy of Membrane Proteins, Monash Institute of Pharmaceutical Sciences, Monash University, Parkville, VIC, Australia.; ^4^ GlycoEra AG, Einsiedlerstrasse 34, 8820 Wädenswil, Switzerland.; ^5^ Department of Microbiology, Biomedicine Discovery Institute, Monash University, Clayton, VIC 3800, Australia.; ^6^ Department of Biochemistry and Pharmacology, Bio21 Molecular Science and Biotechnology Institute, The University of Melbourne, Parkville, VIC, Australia.; ^7^ Gatan Inc., Pleasanton, CA, USA.; ^8^ Department of Biochemistry and Molecular Biology, Monash Biomedicine Discovery Institute, Monash University, Melbourne, VIC, Australia.

## Abstract

High-resolution cryo–electron microscopy (cryo-EM) requires costly 200- to 300-keV cryo–transmission electron microscopes (cryo-TEMs) with field emission gun (FEG) sources, stable columns, constant-powered lenses, autoloader, and direct electron detectors (DED). Recent advances in 100-keV imaging with the emergence of sub–200-keV optimized DED technology promises the development of more affordable cryo-TEMs. So far, 100-keV imaging has required microscopes with FEG sources. We here explored whether a standard 120-keV TEMs with thermionic lanthanum hexaboride (LaB_6_) source can be upgraded with a sub–200-keV DED for high-resolution cryo-EM. Using this imaging configuration, we successfully obtained a 2.65 Å reconstruction for apoferritin, 4.33 Å for 64-kDa hemoglobin, and 4.4 Å for an asymmetric 153kDa membrane protein GPCR. All results were achieved using standard automated data collection with SerialEM, demonstrating the feasibility to collect large cryo-EM datasets with a side-entry cryo-holder. These results showcase a widely accessible solution to obtaining interpretable cryo-EM structures at low cost and contribute to the “democratization” of cryo-EM.

## Teaser

Common 120-keV LaB_6_ TEM enabled with a sub–200-keV optimized detector as an immediate and viable tool for “democratizing” cryo-EM.

## INTRODUCTION

In the past decade, cryo–electron microscopy (cryo-EM) single-particle analysis (SPA) has become a major technique for structural biology (*1*), excelling in areas where protein crystallography has been limited (*2*). The technical infrastructure needed for this endeavor involves specialized cryo-EM microscopes with a high-coherence electron field emission gun (FEG), high operational vacuum for the stability of the FEG, improved columns, constant-power lenses, and specialized cryo-stages. Together, these provide the optical and mechanical stability required for obtaining a typical high-resolution SPA dataset during 12- to 24-hour automated data collection. Given that improvements in electron source, column, and optics were already available for materials sciences, the development of direct electron detectors is likely the most critical innovation in aiding low-dose imaging and achieving the “resolution revolution” in cryo-EM (*3*). Pushing the signal-to-noise level beyond film, originally used in early cryo-EM studies, these detectors were in first instance designed to yield optimal detective quantum efficiency (DQE) for imaging 200- to 300-keV electrons (*4*–*6*). These detectors also made it possible to fractionate the total dose required for a typical exposure (50 to 60 e^−^ Å^−2^) into subframes. This, combined with the advancements in the motion correction algorithm (*7*), facilitated the efficient correction of beam-induced sample drift, as well as residual stage drift in the individual exposures. This made it possible to routinely achieve resolutions below 4 Å for biological specimens (*3*). In addition, many notable innovations in recent years have boosted the achievable resolution in cryo-EM. This includes the incorporation of cold FEGs for cryo-EM (*8*), the development of a stable energy filter to enable zero loss filtering using a narrow slit width of 10 eV or below (*9*), and the improvements in minimizing detector readout noise, namely, correlated double sampling (CDS) mode in Gatan’s K3 detector (*10*) and multiframe CDS implementation in Falcon 3-4 detectors (Thermo Fisher Scientific). Individually or combined, these innovations have made it possible to solve biological structures to sub–2-Å resolution (*11*–*15*).

For most single-particle projects, the total imaging time required for their successful completion can be divided into screening and optimizing sample preparation conditions and the final high-resolution imaging. Screening usually involves assessing grid preparation by imaging multiple grids to identify the conditions that result in optimal particle distribution and quality of ice. During the screening, a limited dataset from two to three grid squares with varying ice thickness is assessed for best particle distribution from each grid. This exercise is also useful to assess potential pitfalls in cryo-EM grid preparation such as preferential orientation and air-water interface or to understand problems arising from biochemistry like intactness of biological complexes and flexibility (*16*–*18*). Ideal cryo-EM samples would typically yield class averages showing multiple orientations as well as secondary structure level details at ~6- to 8-Å resolution. A three-dimensional (3D) reconstruction from such a dataset could potentially yield 3- to 6-Å resolution, enough to screen for complex formation and antibody or fab binding and even for the presence of peptides or small molecules (at ~3- to 4-Å resolution). Once the grid-making conditions have been established from limited processing, the same grid or replicates can be sent for imaging at state-of-the-art 300-keV microscopes such as the TFS Titan Krios or the Jeol Cryo Arm 300 for the collection of large highest-quality datasets (*19*).

The screening process can easily amount for the largest proportion of imaging time required for the successful completion of a project, especially for more challenging samples. It should be noted that higher-quality datasets for screening purposes require relatively expensive cryo–transmission electron microscopes (cryo-TEMs) typically having a 200-keV FEG source, constant-power lenses, direct electron detectors, and often autoloader stages. In this context, lately, potential benefits have been highlighted for performing cryo-EM at 100 keV (*20*) and the application for high-resolution structure determination was shown using a modified DECTRIS EIGER X 0.5-megapixel detector on a TF20 operated at 100 keV (*21*). Since then, a dedicated 100-keV microscope was developed, featuring a 1-megapixel dedicated 100-keV detector (DECTRIS SINGLA), a low chromatic aberration coefficient (C_c_) objective lens, and a Schottky FEG (S-FEG) electron source with compact power supply. The design of this microscope, especially in its nonrequirement of the greenhouse gas sulfur hexafluoride (SF_6_) for insulation, considerably reduced the cost, complexity, and environmental impact (*22*). This system is not yet commercially available, but these studies show the promising future of cheaper microscopy solutions enabling democratization of the field (*16*, *22*, *23*). Taking a lead from these studies, commercial manufacturers have made strides into developing sub–200-keV detectors. This includes the Alpine direct electron detector by Gatan, Falcon-C by Thermo Fisher Scientific, and Quantum C100 from Quantum Detectors. Recently, a microscope by Thermo Fisher Scientific called Tundra has been shown to be an effective tool for sample screening using 100-keV imaging with the Falcon-C detector (*23*). However, this imaging configuration is still a relatively expensive microscope because of its X-FEG source, higher-quality column with a constant-power objective lens, and semiautomated sample loader. One of the main bottlenecks for wide adoption of single-particle cryo-EM hence is the high cost of these imaging capabilities as well as the service contracts to maintain them.

In the context of commercially available sub–200-keV optimized direct electron detectors, we looked for further opportunities for making high-resolution cryo-EM more affordable. We focused especially on the sample screening aspect of cryo-EM SPA, which requires the ability to reach a resolution of 3 to 4 Å in the best-case scenario. However, so far, these detectors have only been tested on costlier FEG source instruments but not yet on standard 120-keV TEMs with a common lanthanum hexaboride (LaB_6_) thermionic source. There are few published cases where LaB_6_ filaments were used to obtain 6- to 10-Å–resolution structures imaged at 400 keV using a Jeol JEM-4000 (*24*–*26*), but these studies predate the introduction of direct electron detectors and were imaged on film. The major inherent factor limiting resolution is the spatial and temporal coherence of the LaB_6_ source. The energy spread for the LaB_6_ source is typically in the range of 1.5 to 2 eV as opposed to <0.8 to 0.9 eV for Schottky FEG (*27*), 0.7 eV for X-FEG, and <0.3 eV for cold FEG (*9*). However, even taking these limitations into account, the line and point resolution of typical 120-keV LaB_6_ TEMs is in the range of 2.0 and 3.6 Å, respectively. In addition, practical limitations such as the substantial sample drift of side-entry cryo-transfer holders, insufficient environmental isolation, and stability in terms of vibration and acoustic noise can further limit the performance. Since dose-fractionated data from a direct electron detector can be used to mitigate problems with stage drift and beam-induced motion, we envisaged that the addition of such a detector on a traditional 120-keV LaB_6_ microscope can hugely boost its performance. For the present study, a standard Tecnai 120-keV LaB_6_ G2 Spirit TWIN microscope (Thermo Fisher Scientific) was retrofitted with Gatan’s sub–200-keV optimized direct electron detector Alpine. At 100 keV, the Alpine detector has been shown to have fourfold better DQE than Gatan’s K3 at the Nyquist frequency (*28*). We characterize this imaging configuration for its utility to derive high-resolution structural data. In addition to well-behaved samples, we show that challenging proteins as well as sub–100-kDa proteins can be resolved to higher resolution using this configuration. This could be a more affordable and widely available option for high-quality cryo-EM SPA for both screening purposes and structure determination.

## RESULTS

### 120-keV LaB_6_ TEM performance aided by a sub–200-keV direct electron detector

To test the impact of a direct electron detector on the performance of a standard 120-keV cryo-capable TEM, we used a Tecnai G2 Spirit with a TWIN objective lens (non–constant-power lens) with a spherical aberration coefficient (C_s_) of 2.2 mm and a Cc of 2.2 mm. The electron source used is a standard LaB_6_ thermionic source with a 15-μm flat tip and a 90° cone angle (DENKA). As these instruments are not typically used for high-resolution data collection, the housing conditions for such a microscope are usually not optimal. With low-DQE charge-coupled device (CCD) cameras traditionally fitted with these microscopes combined with poor vibration isolation and poor stage drift, observing features close to a line resolution of 2.0 Å tends to be difficult but not impossible.

**Fig. 1.**
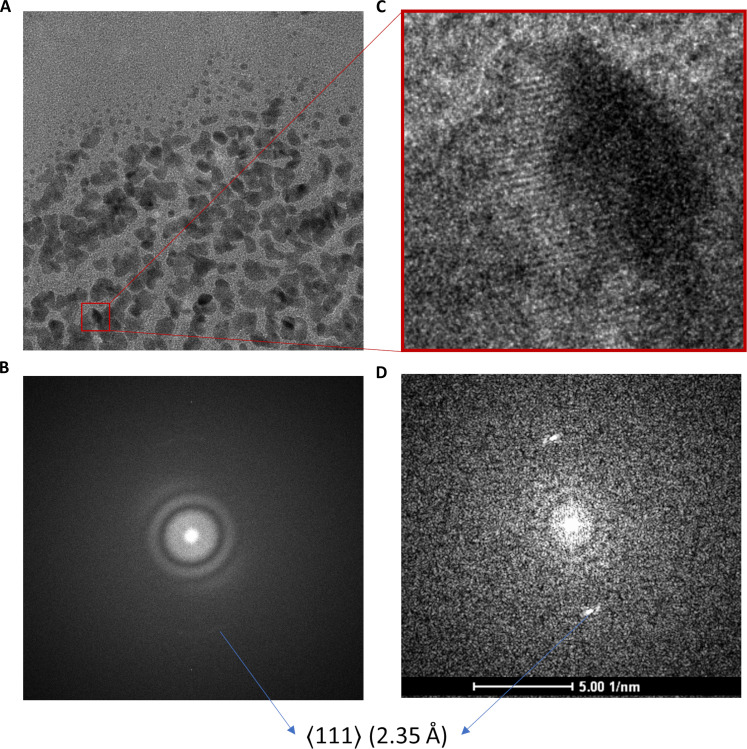
Tecnai G2 Spirit TWIN optical performance with a conventional CCD camera. (**A**) Au cross-grating specimen, 4-s exposure captured on a BM Eagle CCD camera. (**B**) Power spectrum showing washout of information in one direction due to residual stage drift with a weak signal corresponding to ⟨111⟩ reflection corresponding to 2.35 Å seen in the direction least affected by drift. (**C**) Zoomed-in selected area showing Au lattice lines. (**D**) Power spectrum of the selected area (D) showing ⟨111⟩ reflection corresponding to 2.35 Å.

**Fig. 2.**
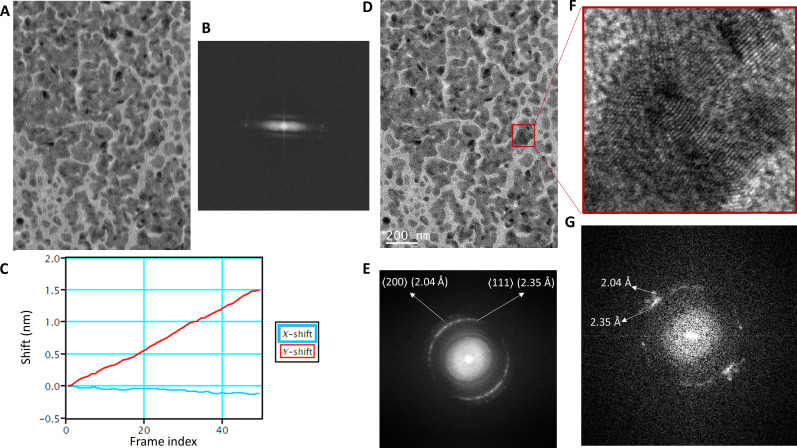
Optical performance of Tecnai G2 Spirit TWIN with an Alpine direct electron detector. (**A**) Summed image of Au cross-grating specimen from 60 fractions of an 8-s exposure taken using a Gatan Alpine camera. (**B**) Power spectrum showing washout of information due to residual stage drift. (**C**) Motion correction in a Gatan digital micrograph showing estimated motion along *x* and *y* axes. (**D**) Motion-corrected average. (**E**) Retrieval of information from motion correction as shown by the power spectrum of the image in (D) showing 2.35- and 2.04-Å rings corresponding to the ⟨111⟩ and ⟨200⟩ reflections, respectively. (**F**) Zoomed-in region from the original image showing the Au lattice and (**G**) the corresponding selected-area power spectrum.

After tuning the microscope for 120 keV, a cross-grating sample mounted on a room-temperature holder was imaged to assess the optical performance of the microscope under parallel illumination conditions. Once the stage drift settled, it was possible to observe a 2.35-Å lattice signal as observed in selected-area fast Fourier transform from a high-resolution transmission electron microscope image captured using a BM Eagle CCD detector (Fig. 1D). The 4-s exposure did result in capturing some residual drift as seen in a directional loss of information in the power spectrum (Fig. 1B). This camera was unmounted and was replaced with the commercially available Gatan sub–200-keV optimized Alpine detector (fig. S1, A to C). As it is a direct electron detector, imaging with the Alpine detector allowed us to save images as dose-fractionated movies, which allows for postacquisition drift correction. Optical alignments of the microscope were checked, and a longer exposure of 8 s was chosen to capture stage drift and environmental vibrations that might affect data collection during typical usage. The resultant exposure showed severe motion blurring due to drift (Fig. 2A), which is also visible in the associated power spectrum (Fig. 2B). The digital micrograph’s built-in motion correction tools were used to analyze and further correct the drift after acquisition. The drift analysis showed that there was ~1.6-nm cumulative drift in the *y* direction and ~0.2-nm drift in the *x* direction. The power spectrum from the motion-corrected aligned average clearly showed the gold ⟨111⟩ (2.35 Å) and ⟨200⟩ (2.04 Å) signals as shown in (Fig. 2, E and G). The same tests were done with a Gatan 626 holder with and without liquid nitrogen (LN_2_) to test for holder performance. In the first instance, we observed washout of Thon rings in the power spectrum caused by vibration (not fixable by drift correction). These vibrations, predominantly acoustic in nature, were seen to have more pronounced impact with cryoholders likely because of the larger surface area associated with LN_2_ dewars compared to the room-temperature holder (fig. S1, D to F). The Alpine camera power supply was identified as the reason for the acoustic vibrations and was relocated to an adjacent utility room, thereby attenuating these vibrations and increasing data quality (fig. S1G). Next, we proceeded to automate the usage of the Alpine camera using SerialEM (*29*) as well as to enable automatic data collection. We performed tests with and without beam image shift on a C-Flat grid (Protochips) to mimic single-particle acquisition for collecting nine holes per stage move. The purpose of this was to test whether switching between lower selected-area (SA) to high SA magnifications required for cycling between hole finding, autofocusing, and data acquisition would lead to any major hysteresis in the non–constant-power TWIN objective lens and/or requires longer beam settling time after image shift. The results of contrast transfer function (CTF) estimation using CTFFIND (*30*) showed that ~90% of the images could be fit to a resolution of 3.5 to 4 Å (fig. S1G) and had an average astigmatism measure of 29.2 nm (fig. S1H). Coma-free alignment is critical for obtaining high-resolution cryo-EM datasets (*31*). In contrast to higher-end Thermo Fisher Scientific microscopes where coma-free alignment is accessible in the user interface, only rotation centering is available on the Tecnai G2 Spirit. This issue is easily mitigated by integrating the camera use with SerialEM (*29*), which makes it possible to access the tilt coils to achieve coma-free alignment as well as to perform coma versus image-shift calibration to reduce astigmatism and coma arising from using beam image shift. Once these calibrations were performed and sufficiently reproducible optical performance was observed, we proceeded to collect SPA datasets.

### Sub–3-Å structure of apoferritin

**Fig. 3.**
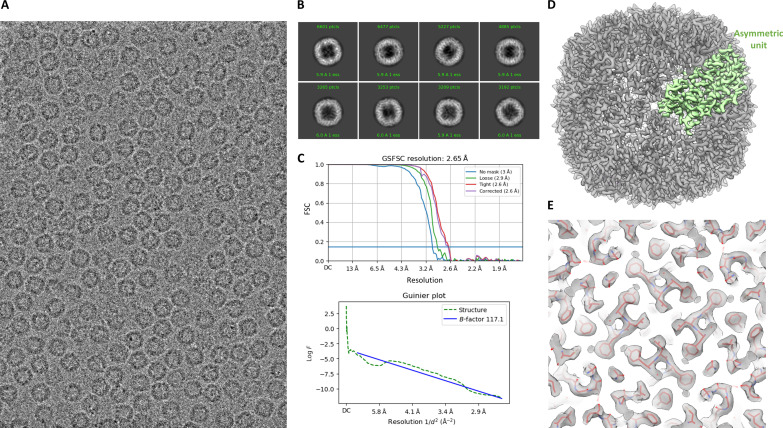
Sub–3-Å structure of apoferritin. (**A**) Representative image of apoferritin at 0.865-μm defocus. (**B**) 2D class averages showing the secondary structure detail. (**C**) FSC plot showing gold standard resolution at 0.143 of 2.65 Å and Guinier plot showing the *B*-factor calculated during refinement in CryoSPARC. GSFSC, gold standard FSC. (**D**) 3D reconstruction of apoferritin at 2.65-Å resolution (gray) and density in green corresponding to one asymmetric unit. (**E**) Map quality showing the well-fitted backbone and side-chain density from rigid-body docking of PDB ID: 7A6A.

**Fig. 4.**
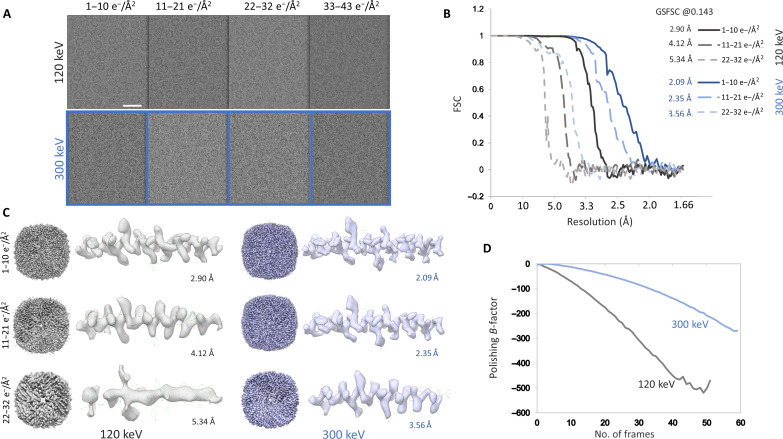
Dose-dependent damage estimation. (**A**) Non–dose-weighted, motion-corrected averages corresponding to every 10 e^−^ Å^−2^, top from the selected area of an apoferritin image taken at 0.865-μm defocus, imaged using Tecnai G2 TWIN operated at 120 keV and captured using a Gatan Alpine camera and bottom corresponding to selected area from the image taken on G1 Titan Krios operated at 300 keV and captured using Gatan K3 at 0.947-μm defocus. (**B**) Gold standard FSC plot for reconstructions from particles for dose range until 32 e^−^ Å^−2^ for both 120 keV (gray) and 300 keV (blue). (**C**) 3D reconstruction for dose range corresponding to the first 32 e^−^ Å^−2^ for both 120 keV (gray) and 300 keV (blue) and zoomed-in region of the map showing α helix comprising residues Phe^38^ to Thr^11^. (**D**) Per-frame *B*-factor calculated during Bayesian polishing in RELION-5 for both 120 keV (gray) and 300 keV (blue).

To assess whether the optical stability seen in a limited number of datasets measured on carbon translates to long data collections required for biological specimen, we used apoferritin as our first test sample. We obtained a throughput of ~170 images/hour with beam image-shift data collection on nine holes and a 15-s stage drift settling time, which was used for four rounds of autofocusing. For the whole dataset, the maximum resolution to which the CTF could be reliably fit using CTFFIND was Gaussian distributed around 4.5 to 5 Å. Further image processing yielded a 2.65-Å final structure (Fig. 3). Although the first 20,000 particles were sufficient to reach 2.92 Å, further addition of particles did not markedly improve the result. The calculated *B*-factor from the Rosenthal and Henderson (*32*) plot was 139 Å^2^, and the sharpening *B*-factor from the Guinier plot calculated during refinement was 117 Å^2^ (Fig. 3D). Although, before data collection, the coma was corrected to a threshold of 0.1 mrad, the average residual coma measured from CTF refinement (*33*) for the nine beam image-shift optics group was noted to be 0.58 and 0.60 mrad in *x* and *y*, respectively. Furthermore, analysis of Bayesian polishing (*34*) *B*-factor (Fig. 4D) showed a steep rise in the estimated *B*-factor as a function of dose. We compared this to polishing *B*-factor obtained for a megapixel-equalized zero loss–filtered dataset of apoferritin acquired on the S-FEG 300-keV Titan Krios captured using a Gatan K3 detector at a pixel size of 0.82 Å. At 120 keV, the low-resolution contrast for non–dose-weighted averages was seen to drop progressively with every accumulated dose of 10 e^−^ Å^−2^. The low-resolution contrast for the 300-keV dataset, albeit being poor when compared to corresponding dose fractions from the 120-keV dataset, followed the same pattern of attenuation with the progression of dose (Fig. 4A). However, for high-resolution information, the resultant reconstructions showed that in the 120-keV dataset compared to the 300-keV dataset, very little high-resolution information is present after 30 e^−^ Å^−2^ (Fig. 4, B and C). These observations are in line with previous studies comparing 100- and 300-keV dose-dependent loss of high-resolution signal in the diffraction pattern from two-dimensional (2D) crystals of C_44_H_90_ paraffin and purple membrane (*20*).

### High-resolution structure of a 153-kDa asymmetric dynamic membrane protein

**Fig. 5.**
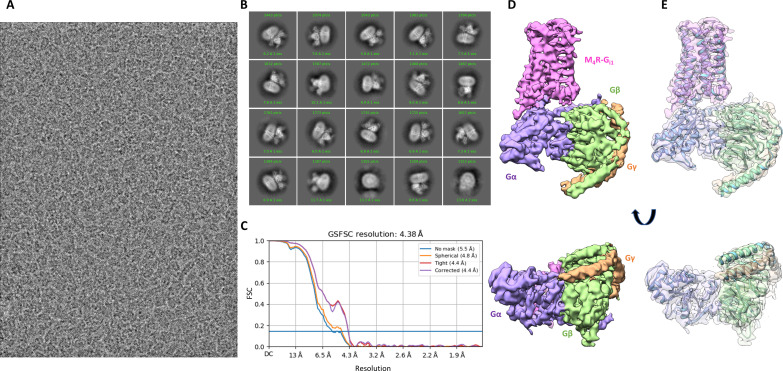
High-resolution structure of a 153-kDa asymmetric dynamic membrane protein. (**A**) Representative image of M4R-G_i1_-Ipx-LY298 at 0.819-μm defocus. (**B**) 2D class averages showing secondary structure detail. (**C**) FSC plot showing gold standard resolution at 0.143 of 4.4 Å. (**D**) 3D reconstruction of M_4_R-G_i1_-Ipx-LY298 at 4.4 Å resolution showing the transmembrane helices of the receptor (purple) and the attached intracellular G protein complex stabilized by scFv16 (masked out). (**E**) PDB ID: 7TRP rigid body docked into the cryo-EM reconstruction.

We further tested the imaging configuration to see how it fares with a more challenging asymmetric sample and its feasibility for screening. For this purpose, we chose the 153-kDa M_4_ muscarinic acetylcholine receptor (M_4_ mAChR) bound to the G_i1_ protein and in complex with the small molecule iperoxo as well as LY298 (M_4_R-G_i1_-Ipx-LY298), which belongs to the group of G protein–coupled receptors (GPCRs). This complex was previously reconstructed to a resolution of 2.4 Å (*35*) from ~6000 zero loss–filtered movies collected at 300 keV on a Titan Krios with a K3 detector in CDS mode. Using the Alpine on the Tecnai G2 Spirit, we collected a megapixel-equalized dataset to match a typical screening dataset that we normally collect on a Talos Arctica. In ~11 hours, we obtained ~1300 movies. Initial analysis using a combination of RELION-4.0 and CryoSPARC ab initio classification followed by nonuniform refinement yielded a resolution of 5.8 Å. Further 3D classification in RELION-5.0 with BLUSH regularization (*36*) followed by refinement using BLUSH regularization yielded a final total of 50,031 particles that reached a resolution of 4.4 Å (Fig. 5). Local resolution estimates (fig. S2F) showed that the resolution in the transmembrane helices (TMs) is of the order of 4.2 to 5 Å with the highest resolution of the order of 4.2 to 4.8 Å for TM3. This resolution in the TMs was sufficient to unambiguously trace the backbone as shown by the rigid-body fit of the previously published M_4_R-G_i1_-Ipx-LY298 structure (fig. S2A). The resolution was sufficient to unambiguously identify a notable tilt in the receptor with respect to the G protein when compared to that solved previously (fig. S2E). The structure had sufficient resolution to identify the presence of the small-molecule (LY298) occupancy in the allosteric binding site, whereas the orthosteric inhibitor iperoxo was not visible (fig. S2D). Notably, in the previously published reconstruction from the 300-keV dataset (*35*), a density for iperoxo was detectable, but the alkyne bond was not visible. Since the 300-keV dataset had 617,793 particles compared to the 50,031 particles in the current study, it is difficult to ascertain whether the lack of the iperoxo density in the structure is due to a poorer signal-to-noise (S/N) ratio of individual particles compared to the zero loss–filtered 300-keV FEG dataset or is a result of partial occupancy or dynamics, which would have benefited from averaging more particles. Although the resolution is not sufficient to unambiguously confirm the identity of LY298, the results show that this resolution level is sufficient to identify the presence or absence of a small ligand at the binding site.

### A 4.33-Å structure of a 64-kDa protein hemoglobin

We further characterized the setup for imaging protein with a molecular mass smaller than 100 kDa. For this purpose, we chose human hemoglobin, a ~64-kDa heterotetrametric heme-containing protein. The data collection strategy was kept similar to that described for the GPCR and apoferritin samples. Hemoglobin particle distribution was less than ideal with more particles present toward the lip of the holes. About 1300 movies were collected between two different grids. Movies with the maximum observable resolution as reported by CTFFIND that exceeded 6 Å were discarded. This resulted in ~700 usable movies. This dataset was sufficient to get 2D class averages with secondary structure–level detail. For the 3D refinement, the conventional ab initio classification with a 30-Å initial search resolution cutoff proved to be intractable to result in a sufficient-quality ab initio model. Instead, an initial low-resolution cutoff of 12 Å and a high-resolution limit of 4 Å proved to be necessary to give a robust ab initio classification result. Particles corresponding to the resultant ab initio class that had secondary structure detail were then further subjected to homogeneous refinement to get a ~6-Å–resolution structure. This structure was used as an initial model for 3D classification using BLUSH regularization in RELION-5.0. Multiple classification rounds were required to home in on a final set of ~8000 particles, which contributed to the final reconstruction with 4.33-Å resolution [gold standard Fourier shell correlation (FSC) (0.143)]. Local resolution estimates (fig. S3D) showed that outer helices in both the α and β chains were exhibiting the lowest resolution of ~5.3 Å, whereas the highest resolution of 4.0 Å was seen for the inner helices (fig. S3, A and D). The density of heme and coordination with His^87^ were also clearly visible as seen by the rigid body–docked Protein Data Bank (PDB) ID: 5NI1 to the EM density (fig. S3, B and C).

## DISCUSSION

Our experiments show that a ubiquitously available 120-keV LaB_6_ electron microscope such as a Tecnai G2 Spirit TWIN can be retrofitted with a sub–200-keV optimized direct electron detector to massively boost its performance level. The cost associated with procuring as well as maintaining these standard 120-keV microscopes is much lower than a modern autoloader system like a Thermo Fisher Scientific Talos Arctica or Glacios or Jeol Cryo Arm 200 or even FEG-based side-entry holder–driven microscopes such as the Talos F200X or Jeol JEM-F200. We were able to successfully integrate the Alpine detector and make it amenable for automated data collection through SerialEM and achieve a working throughput of roughly 170 movies/hour in CDS mode. The 2.65-Å structure of apoferritin achieved using this setup shows that with proper sample preparation, the datasets have enough optical quality to reach a resolution where model building is possible. The results from the Bayesian polishing routine from RELION (*34*) showed that the polishing *B*-factor increases more steeply for the 120-keV dataset compared to the 300-keV dataset (Fig. 4D). We do note here that the 300-keV dataset was collected using zero loss filtering, which is known to increase S/N ratio across the resolution spectrum (*37*). The progressive reduction in high-resolution signal per frame in the 120-keV dataset is likely due to the beam damage caused by the lower-keV electrons combined with a steep CTF envelope owing to poorer coherence of the LaB_6_ source. Since the lower-keV images have very good low-resolution contrast but very little high-resolution information available past 30 e^−^ Å^−2^, the typically used total dose of 50 to 60 e^−^ Å^−2^ for 200- to 300-keV imaging may not be required for 120-keV data collection. The lower dose requirement would further shorten the exposure time required to 3 to 4 s, allowing for an increase in throughput. This would be highly beneficial for challenging asymmetric or flexible targets where increasing the number of particles available for characterization becomes important. These observations are consistent with previous studies (*20*, *21*) where it has been established that 100-keV electrons have more signal-producing elastic scattering as opposed to 300 keV for a given dose. Collecting more data with shorter exposure times at a lower dose is hence a better data collection strategy for imaging at 120 keV. Although the non-CDS mode imaging is not tested in the present study, once used, it should further improve throughput.

The ability of the present imaging configuration to get to a resolution of 4.4 Å for challenging samples like the 153-kDa GPCR (M_4_R-G_i1_-Ipx-LY298) as well as its ability to successfully achieve a 4.33-Å reconstruction for an even smaller 64-kDa protein target like the C2 symmetric human hemoglobin complex, with sparse data, shows the potential of this configuration to achieve below 3- to 4-Å resolution by collecting larger datasets. The resolution achieved in both cases was enough to ascertain the presence or absence of ligands within the complex, especially as seen with the heme complex in the hemoglobin molecule obtained with only 8865 particles (fig. S3). The latest developments in the motion correction algorithm in MotionCor3 as well as RELION-5’s BLUSH regularization technique were critical for 3D classification to glean more information from these sparse datasets. Furthermore, the contrast available at low defocus makes imaging at lower keV an ideal candidate for imaging low–molecular weight protein targets as shown in fig. S4. There is sufficient low-resolution contrast to visually identify particles even at defocus as low as ~0.6 μm for GPCR as well as hemoglobin. The above results show that this imaging configuration is an effective SPA screening tool and, if needed, with larger datasets, can be used to get to a resolution of 3 Å where one can confidently do model building. Samples such as GPCRs, which require screening and characterizing not only on grid square–level variations but also on hole-to-hole–level variations, greatly benefit from screening on TEMs with a multisample loading system. Development of multigrid side-entry cryo-capable holders, which can accommodate three to four grids, would be game changing for using existing 120-keV microscopes for screening purposes. Automated refilling or longer holding times for cryo-conditions to be maintained overnight without the need for manual refill would also allow for longer data collection.

Widely available low-cost 120-keV LaB_6_ microscopes once enabled with an affordable sub–200-keV direct electron detector like the Gatan Alpine detector can massively bring down the economic entry barrier of cryo-EM. This could provide a pathway to cryo-EM, particularly for institutions and countries that currently have no cryo-EM capability. At the same time, dedicated central cryo-EM facilities would receive more optimized samples, allowing for more efficient usage of high-end instrument time. In summary, the upgrade of accessible 120-keV LaB_6_ TEMs could truly democratize cryo-EM with the biggest impact likely on the widespread accessibility of sample screening and optimization.

## MATERIALS AND METHODS

The Alpine detector was bottom mounted as shown in fig. S1 (A and B). The camera was allowed to access the Tecnai G2 Spirit TWIN prespecimen blanker (gun deflector) via a direct connection from TEM lens control electronics to the Gatan camera controller. This allowed the camera to interact with the microscope as a standalone camera. Further integration of the camera with the microscope was performed through SerialEM (*29*) (ver. 4.0.28). Standard SerialEM calibration was carried out to efficiently use the camera along with the microscope.

### Data collection

A Tecnai G2 Spirit TWIN (Thermo Fisher Scientific) equipped with standard LaB_6_ thermionic emission sources (DENKA LaB_6_, 15-μm-radius microflat surface with a 90° cone angle) was operated at 120 keV. A filament heat setting of 27 and a bias setting of 1 were chosen, which resulted in a gun emission of ~4.8 μA. A C2 aperture of 50 μm along with a spot size of 7 was chosen, and the microscope was operated in nanoprobe mode. Diffraction grating replica was used for pixel size calibration in a Gatan digital micrograph. A nominal magnification of 60,700× at camera level corresponding to a calibrated pixel size of 0.4055 Å (super-resolution mode) was chosen for data collection, and the beam was set to satisfy parallel illumination (C2 lens strength ~32.4%). This resulted in a beam with a diameter of ~1.5 μm. The camera was operated in CDS mode. All resultant movies were Fourier binned (2×), dose weighted, and motion corrected using UCSF Motioncor2 (version 1.6.3) (*7*) to output both dose-weighted and non–dose-weighted averages with a resultant pixel size of 0.811 Å. An amplitude contrast of 0.1 was used. The non–dose-weighted averages were used for estimating CTF parameters using CTFFIND 4.1.8 (*30*), using a RELION-4.0.1 (*38*) wrapper.

### Holder and optics stability measurements

Movies were recorded at a dose rate of ~6.5 e^−^ pixel^−1^ s^−1^. A total exposure time of 7 s was used to accumulate a typical SPA dose of 60.4 e^−^ Å^−2^. Automatic data collection using SerialEM was performed to collect a series of test images. A low-magnification map was collected on a relatively flat region of a Au cross-grating (Ted Pella Inc.). Points for image acquisition were set 1 to 2 μm apart for automated data collection to mimic the SPA style data collection routine. A drift settling time of 15 s spent doing autofocusing was used to minimize residual stage drift before data collection. On-axis data collection and beam image-shift data collection using nine holes acquisition per stage shift pattern with and without coma versus image-shift compensation were performed to test for instabilities in imaging that might arise from optics, environmental, or holder instabilities. Data were collected on the same Au cross-grating grid mounted on a room-temperature holder and a Gatan 626 holder at room temperature as well as at −172°C. Before high-resolution data collection, the LN_2_ level was ensured to be below the temperature transfer rod in the cryoholder. Periodic LN_2_ filling of the cryoholder was performed as required, ensuring the above conditions to be met.

### Single-particle data collection

Before imaging all cryo-EM samples, the astigmatism and coma were corrected and coma versus image-shift calibrations were done using SerialEM on a C-Flat grid (Protochips) mounted on a room-temperature holder. Once the beam settings were transferred to SerialEM for acquisition and autofocus setting, the room-temperature holder was removed and the cryo-EM grid was mounted on the stage using a Gatan 626 holder. The filament was kept running during the process.

### Apoferritin

#### Grid preparation

Three microliters of purified apoferritin from Thermo Fisher Scientific (VitroEase Apoferritin Standard) at a concentration of 4 mg/ml was applied on UltraAuFoil (*39*) R 1.2/1.3 300 mesh. The grids were glow discharged for 30 s at 30 mA in an atmosphere before application of the sample. The excess protein solution was blotted off using a blot force of −1 and a blot time of 3 s in a Mark IV Vitrobot (Thermo Fisher Scientific) at 100% humidity and 4°C.

#### Data collection

Beam image-shift data collection with nine holes acquisition per stage shift was performed with coma compensation on. Movies were collected with a total dose of 50.3 e^−^ Å^−2^ accumulated over 6.5-s exposure time at a dose rate of 5.25 e^−^ pixel^−1^ s^−1^ fractionated into 52 frames. A total of 923 movies were collected in a time frame of 5.45 hours.

#### Image processing

Particle picking was performed using Gautomatch (0.53) (https://github.com/JackZhang-Lab/Gautmatch/) on the first 100 images and extracted and binned four times using RELION-4.0.1. These particles were then subjected to 2D classification followed by ab initio and 3D refinement in CryoSPARC 4.2.0 (*40*). The coordinates were then exported back to RELION-4.0.1 using pyem v0.5 and re-extracted in RELION-4.0.1 centered on refined coordinates. The particles were then used for training using Topaz picker (*41*), and the trained model was used for particle picking on the full dataset. The resultant particles were then extracted and binned four times and were imported to CryoSPARC 4.2.0 (*40*). After 2D classification and 3D homogeneous refinement, the final set of particles (127,118) was arrived upon (Table 1). This final set of particles was then extracted similarly as described above at native pixel size and was further processed in CryoSPARC 4.2.0 (*40*). The refined particles were then subjected to Bayesian polishing in RELION-4.0.1 and were then further processed in CryoSPARC 4.2.0 (*40*). Several rounds of heterogeneous refinement to exclude noisy particles and CTF refinement yielded a 2.65-Å map (gold standard FSC 0.143 criteria). The pixel size calibration was verified compared to PDB ID: 7A6A.

**Table 1.** Cryo-EM data collection, refinement, and validation statistics.

Data collection and processing	#1 Apoferritin (EMDB-44745)	#2 M_4_R-G_i1_-Ipx-LY298 (EMB-44343)	#2 Hemoglobin (EMDB-44746)
Magnification	60,700×	60,700×	60,700×
Voltage (kV)	120	120	120
Dose rate (e^−^ pixel^−1^ s^−1^)	5.25	6.74	5.07
Electron exposure (e^−^ Å^−2^)	50.3	60.4	55
Defocus range (μm)	0.4–0.8	0.5–1.0	0.5–1.2
Super-resolution pixel size (Å)	0.4055	0.4055	0.4055
Number of movies	923	1324	508 + 860
Number of frames	52	61	55
Time taken (hours)	5.45	~11	~11
Symmetry imposed	O	C1	C2
Initial particle images (*n*)	387,893	479,634	382,710
Final particle images (*n*)	127,118	50,089	8865
Map resolution (Å)	2.65	4.4	4.33
Gold standard FSC threshold	0.143	0.143	0.143
Sharpening *B*-factor (Å^2^)	−117.1	−151.9	−194.9

### Dose damage estimation

#### 300-keV data collection

Apoferritin was frozen as mentioned above, and data were collected using a G1 Krios (Thermo Fisher Scientific) operated at 300 keV, at a C2 aperture of 50 μm, and in energy-filtered TEM mode with imaging done on a Gatan K3 direct electron detector equipped with a Gatan BioQuantum energy filter. Imaging was performed at a nominal magnification of 105,000×, with zero loss filtering done using a 10-eV slit width. The K3 was operated in CDS mode with an effective pixel size of 0.82 Å. A dose rate of 8.9 e^−^ pixel^−1^ s^−1^ was used to accumulate a total dose of 60.21 e^−^ Å^−2^ over 5.0-s exposure time; this dose was further fractionated into 60 frames. Automatic data collection using beam image shift was performed using EPU software (Thermo Fisher Scientific).

Processing was done as mentioned above with the only exception that further refinement using shiny particles from the Bayesian polishing step was not performed. A final particle set of 106,568 particles resulted in 1.89 Å (gold standard FSC 0.143 criteria).

#### Image processing

Both the 120- and 300-keV final sets of particles were re-extracted from non–dose-weighted motion-corrected averages corresponding to every 10 e^−^ Å^−2^ increment. The dose-limited re-extracted particles were autorefined with their respective full dose final reconstructions as the initial model. Initial low-pass filtering for refinement was restricted to 12 Å. The estimated resolution from each dose-limited particle set along with its corresponding FSC (gold standard 0.143 criteria) plot is shown in Fig. 4B.

### M_4_R-G_i1_-Ipx-LY298

#### Grid preparation

Three microliters of M_4_R-G_i1_-Ipx-LY298 purified as described previously (*35*) was applied on UltraAuFoil R 1.2/1.3 300 mesh at a concentration of 15 mg/ml. The grids were glow discharged for 180 s at 15 mA in an atmosphere before application of the sample. The excess protein solution was blotted off using a blot force of 10 and a blot time of 2.5 s in a Mark IV Vitrobot (Thermo Fisher Scientific) at 100% humidity and 4°C.

#### Imaging

Beam image-shift data collection with nine holes acquisition per stage shift was performed with coma versus image shift–calibrated beam tilt compensation on. Data were collected in CDS mode with a super-resolution pixel size of 0.4055 Å. Movies were collected with a total dose of 60.53 e^−^ Å^−2^ accumulated over 6.09-s exposure time at a dose rate of 6.74 e^−^ pixel^−1^ s^−1^ fractionated into 52 frames. A total of 1324 movies were collected in a time frame of ~11 hours.

#### Image processing

The resultant movies were Fourier binned (2×), dose weighted, and motion corrected using UCSF Motioncor3 (*7*) to output both dose-weighted and non–dose-weighted averages with a resultant pixel size of 0.811 Å. Particle picking was performed using Gautomatch (0.53) on the first 100 images and extracted and binned four times. These particles were then subjected to 2D classification followed by ab initio and 3D refinement in CryoSPARC 4.2.0. The coordinates were then exported back to RELION using pyem v0.5 (*42*) and re-extracted in RELION-5.0 centered on refined coordinates. The particles were then used for training using Topaz picker (*41*) through a RELION-5.0 wrapper, and the trained model was used for particle picking on the full dataset. This resulted in 479,634 particles, which were then extracted and binned four times and were imported to CryoSPARC 4.2.0. After 2D classification and 3D homogeneous refinement, the final set of 75,816 particles was arrived upon (Table 1). This final set of particles was then re-extracted similarly as described above but with binning two times and was further processed in CryoSPARC 4.2.0. The refined particles were then subjected to Bayesian polishing in RELION-5.0 and were then reimported and further processed in CryoSPARC 4.2.0. Several rounds of heterogeneous refinement to exclude noisy particles and nonuniform refinement (*43*) yielded a 5.36-Å map (gold standard FSC 0.143 criteria).

The volume erase tool in UCSF Chimera (*44*) was used to remove the micelle density from the map, and a mask containing only the TM region and the G protein was prepared from this map in RELION-5.0 and was used for further 3D classification with BLUSH regularization on the full dataset. The highest-resolution class corresponding to 50,089 particles was then re-extracted at native pixel size and subjected to mask refinement with BLUSH regularization. This pushed the resolution to 4.8 Å (gold standard FSC 0.143 criteria). Polishing followed by per-particle defocus refinement further improved the map to achieve 4.38-Å resolution (gold standard FSC 0.143 criteria) (Fig. 5C).

### Hemoglobin

#### Protein purification

Hemoglobin was purified from human blood collected from a healthy adult volunteer. Before the collection of blood, informed consent of the volunteer was obtained in accordance with 2022-30658-70864 approved by the Monash University Human Research Ethics Committee. Erythrocytes were isolated via centrifugation, and cell pellets were diluted in lysis buffer (50 mM tris and 200 mM NaCl, pH 7.4). To isolate hemoglobin, erythrocytes were lysed with a tight-fit dounce homogenizer and clarified by centrifugation at 18,000 rpm. Cell lysate was further purified using a Superdex 26/600 S200 size exclusion column (Cytiva) pre-equilibrated in size exclusion chromatography buffer (50 mM tris and 200 mM NaCl, pH 7.4). Fractions were collected, pooled, and concentrated to ~10 mg/ml using a 30-kDa spin filter column (Millipore) before being stored at −80°C.

#### Grid preparation

Three microliters of purified hemoglobin sample at a concentration of 10 mg/ml was applied on UltraAuFoil R 1.2/1.3 300 mesh. The grids were glow discharged for 30 s at 30 mA in atmosphere before application of the sample. The excess protein solution was blotted off using a blot force of −1 and a blot time of 3 s in a Mark IV Vitrobot (Thermo Fisher Scientific) at 100% humidity and 4°C.

#### Imaging

Beam image-shift data collection with nine holes acquisition per stage shift was performed with coma versus image shift–calibrated beam tilt compensation enabled. Data were collected in CDS mode with a super-resolution pixel size of 0.4055 Å. Movies were collected with a total dose of 50.14 e^−^ Å^−2^ accumulated over 6.711-s exposure time at a dose rate of 5.07 e^−^ pixel^−1^ s^−1^ fractionated into 55 frames. A total of 1368 movies between two grids were collected.

#### Image processing

**Fig. 6.**
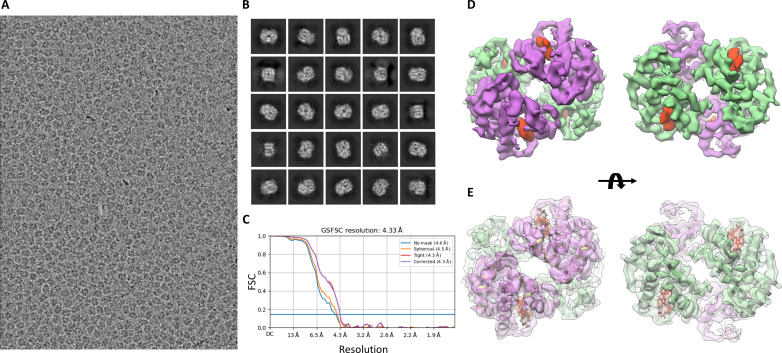
High-resolution structure of a 64-kDa protein hemoglobin. (**A**) Image of human hemoglobin at 0.853-μm defocus. (**B**) 2D class averages showing secondary structure detail. (**C**) Resolution according to gold standard FSC at 0.143 of 4.33 Å. (**D**) C2 symmetric 3D reconstruction of human hemoglobin with purple density showing an α chain, green showing a β chain, and heme density in red. (**E**) PDB ID: 5NI1 rigid body docked into the cryo-EM reconstruction.

The resultant movies were Fourier binned (2×), dose weighted, and motion corrected using UCSF Motioncor3 (*7*) to output both dose-weighted and non–dose-weighted average with a resultant pixel size of 0.811 Å. Particle picking was performed using Gautomatch (0.53) on the first 100 images and extracted and binned four times. These particles were then subjected to 2D classification followed by ab initio and 3D refinement in CryoSPARC 4.2.0. The coordinates were then exported back to RELION using pyem v0.5 and re-extracted in RELION-5.0 centered on refined coordinates. The particles were then used for training using Topaz picker through RELION-5.0, and the trained model was used for particle picking on the full dataset. This resulted in 382,710 particles. In CryoSPARC 4.2.0, ab initio classification was performed using an initial low-resolution search cutoff of 12 Å, a high-resolution limit of 4 Å, and three classes with class similarity set to 0. Particles corresponding to the resultant ab initio class that had secondary structure detail were then further subjected to homogeneous refinement to get a ~6-Å–resolution structure. This structure was used as an initial model for 3D classification using BLUSH regularization in RELION-5.0. Multiple classification rounds were done to reach a final set of 8865 particles (Table 1), which contributed to the final reconstruction with 4.33-Å resolution (gold standard FSC 0.143) (Fig. 6C). Visualization and rigid-body docking for all structures were performed using UCSF Chimera (*44*) (ver. 1.16).
