# Supplementary material for: High-resolution cryo-EM using a common LaB6 120-keV electron microscope equipped with a sub–200-keV direct electron detector
Source: Sci Adv. 2025 Jan 3;11(1):eadr0438. doi: 10.1126/sciadv.adr0438 (PMC11698077; doi:10.1126/sciadv.adr0438)
Supplement: Supplementary file 1 — Figs. S1 to S4 [file sciadv.adr0438_sm.pdf]

Science Advances
AAAS

Supplementary Materials for

High-resolution cryo-EM using a common LaB_6 120-keV electron microscope equipped with a sub–200-keV direct electron detector

Hariprasad Venugopal *et al.*

Corresponding author: Hariprasad Venugopal, hari.venugopal@monash.edu;
Georg Ramm, georg.ramm@monash.edu

Sci. Adv. **11**, eadr0438 (2025)
DOI: 10.1126/sciadv.adr0438

This PDF file includes:

Figs. S1 to S4

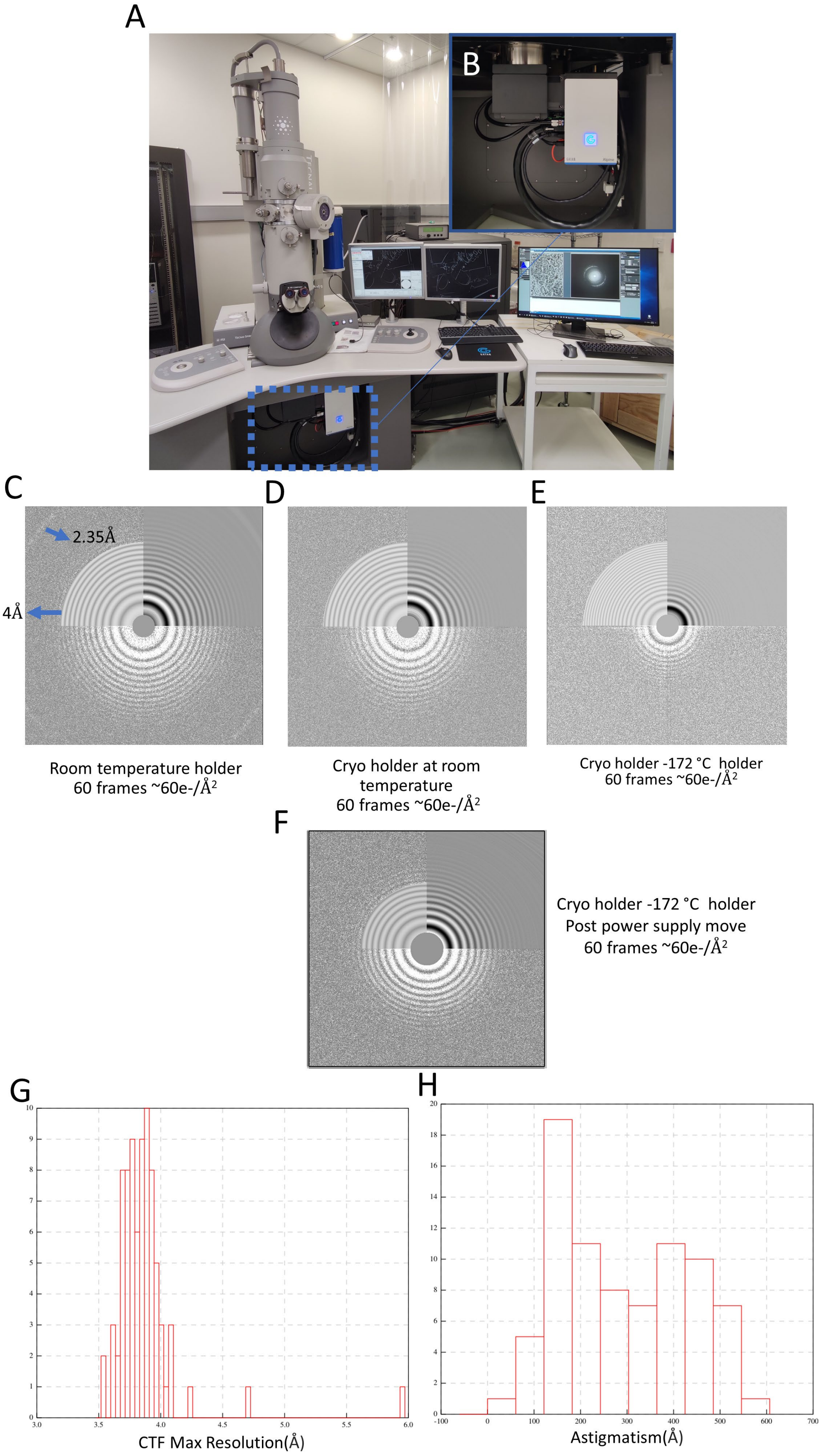
A
B
C
2.35Å
4Å
D
E
Room temperature holder
60 frames ~60e-/Å²
Cryo holder at room temperature
60 frames ~60e-/Å²
Cryo holder -172 °C holder
60 frames ~60e-/Å²
F
Cryo holder -172 °C holder
Post power supply move
60 frames ~60e-/Å²
G
CTF Max Resolution(Å)
H
Astigmatism(Å)

Fig. S1. Tecnai G2 Spirit integration with GATAN Alpine detector. **(A)** Tecnai G2 TWIN 120-keV LaB6 microscope retrofitted with **(B)** GATAN Alpine direct electron detector. **(C)** Power-spectrum from Au cross grating image taken mounted on room temperature holder (top left quadrant showing CTFFIND fit till 4 Å and the top right quadrant with simulated fit), **(D)** from sample mounted on cryoholder but at room temperature showing loss of high-resolution signal **(E)** from image of sample mounted on cryoholder at cryo temperature showing uncorrectable vibration. **(F)** Power-spectrum from c-flat holey carbon grid post power supply move. **(G)** Astigmatism and **(H)** max CTF-max-resolution histogram from 80 movies collected using beam image-shift data collection.

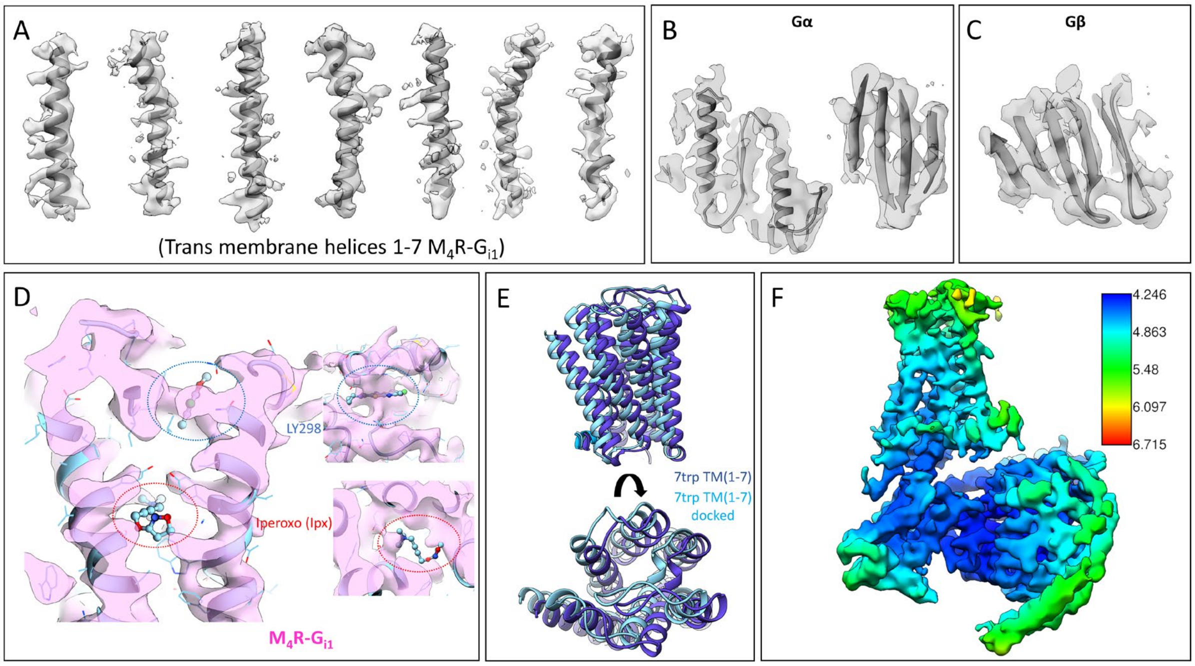

Fig. S2. Map quality of M4R-G_{i1}-Ipx-LY298 complex. (A)Cryo-EM density of transmembrane helices of M_4R-G_{i1} rigid body docked with pdb id: 7TRP. (B) cryo-EM density from G alpha showing alpha helices with sidechain density as well as beta-sheet density. (C) Beta sheets from G beta. (D) M_4R-G_{i1} region binding to ligand LY298 on the top density encompassed in (blue dotted line) and iperoxo missing density as shown by red dotted lines. (E) PDB id: 7TRP TMs rigid body docked to cryo-EM density and aligned with undocked pdb showing tilt with respect to the published structure. (F) Local resolution map showing differential resolution within M_4R-G_{i1}-Ipx-LY298 map.

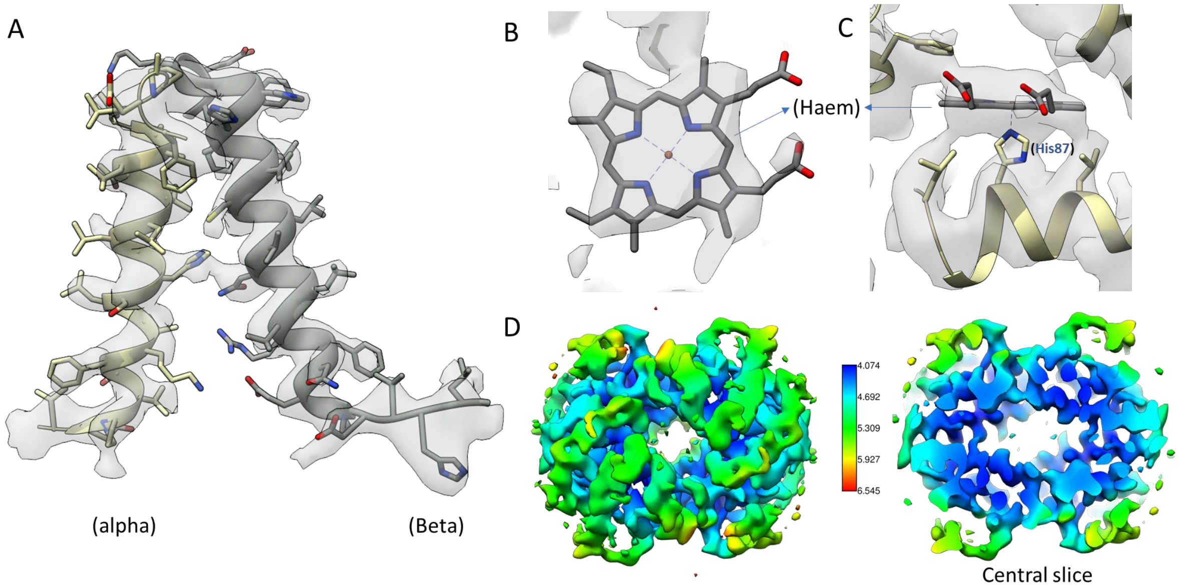

Fig. S3. Map quality haemoglobin (pdb id: 5NI1 rigid body docked into the cryo-EM reconstruction): **(A)** Region comprising residue Val93-His117 of alpha chain and region comprising residue His97-Glu121 of the beta chain of Haemoglobin reconstruction. **(B)** Haem density and **(C)** Density showing haem and His87 coordination **(D)** Map coloured according to local resolution.

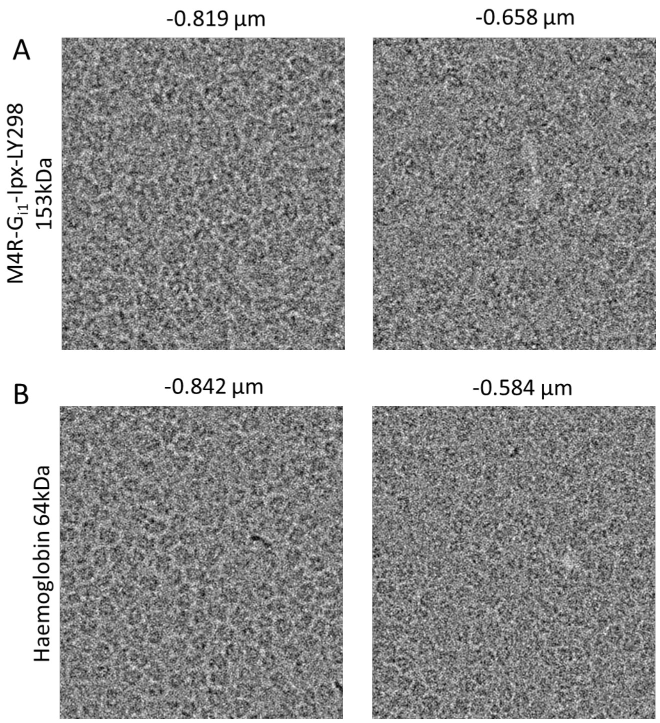

Fig. S4. Contrast at low defocus for sub 200 kDa molecules. **(A)** M4R-G_{i1}-Ipx-LY298 and **(B)** Haemoglobin collected on UltrAUfoil grids at different defocus (indicated above each image) showing contrast to locate particles even in images acquired with lower than a micron defocus.
